# Equilibria Between Cell Membranes and Electrolyte Solution: Effect of Fatal Accidental Hypothermia

**DOI:** 10.1007/s00232-016-9875-4

**Published:** 2016-02-03

**Authors:** Aneta D. Petelska, Joanna Kotyńska, Monika Naumowicz, Zbigniew A. Figaszewski

**Affiliations:** Institute of Chemistry, University in Bialystok, K. Ciolkowskiego 1K, 15-245 Białystok, Poland

**Keywords:** Fatal accidental hypothermia, Surface charge density, Erythrocytes, Thrombocytes, Microelectrophoresis, Acid–base equilibria

## Abstract

Equilibria between the membranes of erythrocytes as well as thrombocytes and solution ions in fatal accidental hypothermia were analyzed using a theoretical four-equilibria model. The model was developed to determinate parameters characterizing cell membrane—surrounding ion interactions: the total surface concentrations of both acidic and basic groups *C*_A_, *C*_B_, and association constants *K*_AH_, *K*_BOH_. Knowledge of these parameters was necessary to calculate the theoretical values of surface charge density. The model was validated by curve-fitting the experimental data points to simulated data generated by the model. The experimental and theoretical surface charge density values agree at pH 2–8, at higher pH the deviation was observed.

## Introduction

Biological membranes are complex heterogeneous nanostructures containing a variety of compounds. Lipids are the major ones. Membranes are formed from lipid bilayers in which the other compounds such as proteins, carbohydrates, and vitamins are immersed or bounded. Biological membranes carry electric charges of different origin. Part of the charge fixed to the membrane originates from dissociation of the carboxyl groups of sialic acids. Other negative charges stem from several phospholipids. Proteins also, with their cationic and anionic groups, contribute to the charge on the membrane. Protons are the charge-determining ions for all these groups, and the membrane charge density is therefore directly dependent on the pH. At essentially all physiological pH values, the net membrane charge is negative reaching values of a few to a few tens of μC cm^−2^ and natural membranes have isoelectric point around pH 4 (Nalecz and Wojtczak 1982; Norde 2011). The membrane surface charge density is an important parameter for characterizing equilibria in the membranes. This parameter strongly depends on environmental conditions such as pH and membrane composition. Changes of the membrane surface charge density values are observed in vitro [adding ionizable surfactants (McLaughlin and Harary 1976), altering the membrane composition] as well as in vivo [sudden unexpected death (Kotyńska et al. 2012), fatal carbon monoxide poisoning (Szeremeta et al. 2013; Petelska et al. 2015), fatal accidental hypothermia (Szeremeta et al. 2015)] conditions.

Hypothermia is defined as an unintentional decline in the core temperature below 35 °C. At this temperature, the coordinated systems responsible for thermoregulation begin to fail. Accidental hypothermia refers to a spontaneous reduction of core body temperature, usually as a result of exposure to cold environments without adequate protection (Hanania and Zimmerman 1999). The postmortem diagnosis of hypothermia remains difficult to ascertain today, despite progress made during the past several decades in the realm of forensic pathology (Palmiere et al. 2013). Important findings for the diagnosis of hypothermia-related death such as frostbites, hemorrhagic spots of the gastric mucosa (Wiszniewski’s spots), cardiomyocyte necrosis do not appear in all cases. When there are poor morphological evidence, researchers intensively search for reliable biochemical and physicochemical parameters that would help to determine the cause of death from hypothermia (Jakubeniene et al. 2009).

The present investigation was undertaken to evaluate the usefulness of physicochemical measurements in diagnosis of fatal hypothermia. Studies of electrical properties of human cell membranes have been realized recently by Petelska et al. (Kotyńska et al. 2012; Petelska et al. 2012; Szeremeta et al. 2013, 2015). Our last paper (Szeremeta et al. 2015) presents experimental surface charge density values of blood cells in fatal accidental hypothermia, obtained by microelectrophoresis method. Due to the lack of published studies concerning the effect of hypothermia on equilibria between cell membranes and surrounding ions, in this paper, we provide a quantitative description of the equilibria. We validated the theoretical values (obtained from equations presented in Methods section) with experimental ones. Data presented in this work, obtained as a result of mathematical derivations and confirmed experimentally, in our opinion, can help in better understanding the effect of fatal accidental hypothermia on biological membrane surface properties.

## Materials and Methods

### Materials

Approval for this study was granted by the Ethics Review Board of Medical University of Bialystok (No. R-I-002/533/2010). Blood (pH 6.6) was obtained from sober individuals during autopsies made at the Forensic Medicine Department at the Medical University of Bialystok. The subject of the examination was based on 10 selective fatal hypothermia (five men and five women, mean age 43.7 years, range 23–71) autopsied in the year 2013. All blood samples were routinely obtained from the femoral vein and put into chemically and biologically clean glass containers. The donated samples were comparatively analyzed to the control samples taken from live individuals from the Blood-service Centre in Bialystok. The control group was made up of 15 honorary blood donors (seven men and eight women, mean age 35.3 years, range 24–42). The samples were transferred to the laboratory (Department of Electrochemistry, Institute of Chemistry, University of Bialystok) immediately after performing the autopsies. All autopsies were performed within 24 h after death. The whole analyses were done directly after receiving of the samples.

#### Preparation of Erythrocytes from Blood

Erythrocytes were isolated from 2 ml of liquid whole blood by centrifugation at 900×*g* for 8 min at room temperature. The supernatant thrombocyte-rich plasma was removed and saved for subsequent processing while the erythrocytes were washed three times with isotonic saline (0.9 % NaCl) at 3000 g for 15 min. After the final wash, the erythrocyte pellet was resuspended in isotonic saline for electrophoretic measurement.

#### Preparation of Thrombocytes from Plasma

The thrombocyte-rich plasma was centrifuged at 4000×*g* for 8 min. The supernatant plasma was removed and discarded. The thrombocyte pellet was washed three times with isotonic saline by centrifugation at 3000 g for 15 min. After the final wash, the thrombocytes were resuspended in isotonic saline for electrophoretic measurement.

All solutions and cleaning procedures were performed with water purified using a Milli—Qll system (18.2, Millipore, USA).

### Methods

#### Microelectrophoretic Mobility Measurements

The electrophoretic mobility of blood cells was obtained by performing an electrophoresis experiment on the sample and measuring the velocity of the particles using Laser-Doppler Velocimetry (LDV) with the Zetasizer Nano ZS (Malvern Instruments, UK). The measurements were carried out as function of pH (from 2 to 11), using 0.9 % NaCl as a supporting electrolyte. The cell membranes were suspended in NaCl solution and titrated to the desired pH using HCl or NaOH. The reported values represent the average of at least six measurements performed at a given pH.

#### Experimental Surface Charge Density Determination

The experimental surface charge density values were determined from electrophoretic mobility measurements using the Eq. 1 presented below (Alexander and Johnson 1949):

1 $$\updelta = \frac{\eta \cdot u}{d},$$

where *η* is the viscosity of solution, *u* is the electrophoretic mobility, and *d* is the diffuse layer thickness (Eq. 2, Barrow 1996):

2 $$d = \sqrt {\frac{{\varepsilon \cdot \varepsilon_{0} \cdot R \cdot T}}{{2 \cdot F^{2} \cdot I}}} ,$$

where *R* is the gas constant, *T* is the temperature, *F* is the Faraday number, *I* is the ionic strength of 0.9 % NaCl, and *εε*_o_ is the permeability of the electric medium.

#### Theoretical Surface Charge Density Determination

The dependence of the surface charge density of blood cell membranes on the pH of the electrolyte solution can be mathematically described. This model assumes the existence of four equilibria. Two of these equilibria are associated with positive groups (for example: phospholipids or proteins) and with hydroxide and chloride ions. The other two equilibria are associated with negative species of phospholipids or proteins and sodium and hydrogen ions. The model is presented in full detail in previous papers (Dobrzyńska et al. 2006; Kotyńska et al. 2012; Petelska et al. 2015).

Solving a system of equations yields the final equations:

- equation describing surface charge density of the cell membrane:
  3 $$\frac{\delta }{F} = \frac{{C_{\text{B}} }}{{1 + K_{\text{BOH}} a_{{{\text{OH}}^{ - } }} + K_{\text{BCl}} a_{{{\text{Cl}}^{ - } }} }} - \frac{{C_{\text{A}} }}{{1 + K_{\text{AH}} a_{{{\text{H}}^{ + } }} + K_{\text{ANa}} a_{{{\text{Na}}^{ + } }} }}$$
- linear equations obtained by adequate simplifications (for high and low concentration of hydrogen ions)

4 $$\frac{{\delta a_{{{\text{H}}^{ + } }} }}{F} = \frac{{C_{\text{B}} }}{{1 + K_{\text{BCl}} a_{{{\text{Cl}}^{ - } }} }}a_{{{\text{H}}^{ + } }} - \left( {\frac{{C_{\text{B}} K_{\text{BOH}} K_{\text{W}} }}{{(1 + K_{\text{BCl}} a_{{{\text{Cl}}^{ - } }} )^{2} }} + \frac{{C_{\text{A}} }}{{K_{\text{AH}} }}} \right)$$

5 $$\frac{\delta }{{{\text{Fa}}_{{{\text{H}}^{ + } }} }} = - \left( {\frac{{C_{\text{A}} }}{{1 + K_{\text{ANa}} a_{{{\text{Na}}^{ + } }} }}} \right)\frac{1}{{a_{{{\text{H}}^{ + } }} }} + \left( {\frac{{C_{\text{B}} }}{{K_{\text{BOH}} K_{\text{W}} }} + \frac{{C_{\text{A}} K_{\text{AH}} }}{{(1 + K_{\text{ANa}} a_{{{\text{Na}}^{ + } }} )^{2} }}} \right),$$

where $$\delta$$ is the surface charge density, $$a_{{{\text{H}}^{ + } }}$$, $$a_{{{\text{Na}}^{ + } }}$$, $$a_{{{\text{OH}}^{ - } }}$$, $$a_{{{\text{Cl}}^{ - } }}$$ is the volume concentrations of solution ions; $$C_{\text{A}}$$ is the total surface concentration of the membrane acidic groups; $$C_{\text{B}}$$ is the total surface concentration of the membrane basic groups, $$K_{\text{AH}}$$, $$K_{\text{ANa}}$$, $$K_{\text{BOH}}$$, $$K_{\text{BCl}}$$—association constants, $$F = 96487$$$$\left[ {\frac{C}{\text{mol}}} \right]$$ is the Faraday constant.

The model enabled the calculation of $c_{\text{A}}$, $c_{\text{B}}$, $K_{\text{AH}},$ and $K_{\text{BOH}}$ values from Eqs. 4 and 5. Determination of all above parameters was possible by making an assumption that the $K_{\text{ANa}}$ and $K_{\text{BCl}}$ association constants values are the same as those obtained for phosphatidylcholine liposomes, which are equal to 0.230 and 0.076 (m^3^/mol), respectively (Dobrzyńska et al. 2007). Calculated $c_{\text{A}}$, $c_{\text{B}}$, $K_{\text{AH}},$ and $K_{\text{BOH}}$ values were substituted into Eq. 3 to obtain surface charge densities' theoretical data for erythrocytes and thrombocytes membranes in fatal accidental hypothermia.

## Results and Discussion

Data presented in the paper are based on our previous publication (Szeremeta et al. 2015), in which there are only experimental results of the surface charge density changes of the blood cell membranes after fatal accidental hypothermia. However, this work presents the theoretical approach for describing equilibria occurring in the erythrocytes and thrombocytes membranes as well as determination of parameters characterizing the equilibria. The theoretical values of surface charge densities after fatal accidental hypothermia were determined from Eq. 3 (“Materials and Methods” section).

Biological membranes are very complicated structures. For that reason they are difficult to analyze by theoretical considerations. There are a lot of equilibria existing between the membrane components, as well as between them and surroundings. Since it is not possible to define values of each parameters characterizing the equilibria, it was assumed an adequate number of parameters that would include the mean values for all equilibria. Knowledge of parameters such as total surface concentration of membrane acidic ($c_{\text{A}}$) and basic ($c_{\text{B}}$) groups on the erythrocyte as well as thrombocyte surface and their average association constants with hydrogen ($K_{\text{AH}}$) and hydroxyl ($K_{\text{BOH}}$) ions may be important in describing the multiple processes involving membranes in the living cell. One of such processes is fatal accidental hypothermia which is associated with a number of potentially serious physiological changes and destructive processes at the cellular level. The interactions between biological membrane components and electrolyte solution ions were previously described by the four-equilibrium mathematical model (Dobrzyńska et al. 2006).

**Fig. 1:**
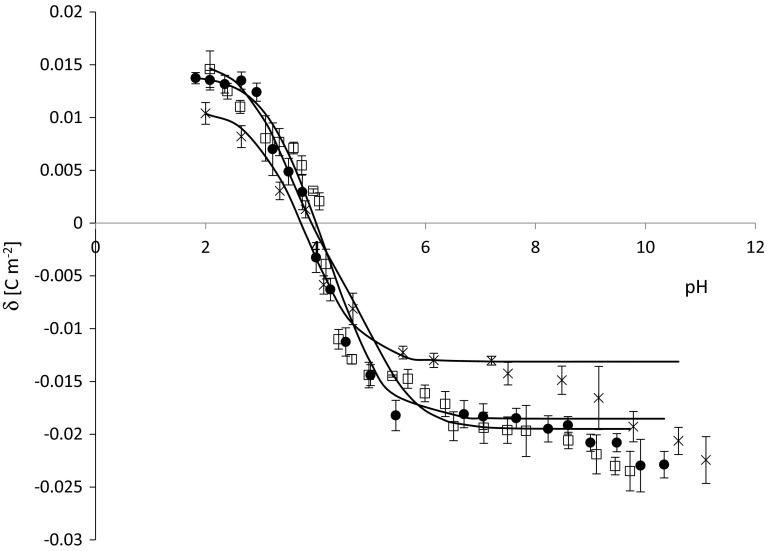
Surface charge density of erythrocytes membrane as a function of the pH of the electrolyte solution: *times*—control, *black circle*—sudden unexpected death, *white square*—fatal accidental hypothermia. Points denote experimental values. Continuous line links the theoretical values

**Fig. 2:**
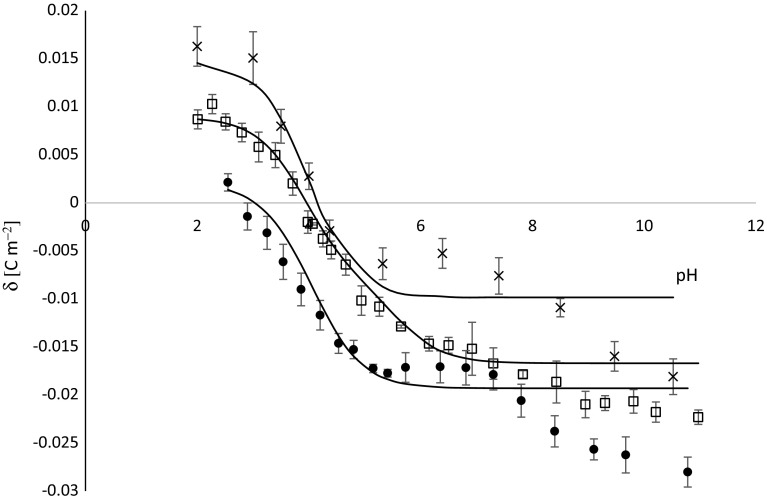
Surface charge density of thrombocytes membrane as a function of the pH of the electrolyte solution: *times*—control, *black circle—*sudden unexpected death, *white square*—fatal accidental hypothermia. Points denote experimental values. Continuous line links the theoretical values

The surface charge densities of the control (Kotyńska et al. 2012), sudden unexpected death (Kotyńska et al. 2012), and fatal accidental hypothermia erythrocytes and thrombocytes are plotted as a function of pH in Figs. 1 and 2, respectively. The figures contain experimental (points) and theoretical (continuous lines) data for comparison of their conformity. As it can be seen from Fig. 1 fatal accidental hypothermia causes an increase of surface charge density values in whole pH range compared with control membrane. Fatal accidental hypothermia and sudden unexpected death surface charge density values are similar. In the case of thrombocytes (Fig. 2), it can be noted that fatal accidental hypothermia surface charge density curve is located between control and sudden unexpected death curves. As it is presented in Figs. 1 and 2, good fits were obtained between the experimental and theoretical data in the pH range 2–8, above pH >8 the theoretical curves differ from the experimental points. The deviation from the theoretical curve may be caused by the interactions which probably exist in our system but which were not considered by the theoretical model.

Tables 1 and 2 present the parameters ($c_{\text{A}}$, $c_{\text{B}}$, $K_{\text{AH}}$, $K_{\text{BOH}}$) characterizing erythrocyte and thrombocyte surfaces, respectively. These data were analyzed using standard statistical analysis and are expressed as mean standard deviation. The physicochemical parameters characterizing both erythrocyte and thrombocyte surfaces ($c_{\text{A}}$, $c_{\text{B}}$, $K_{\text{AH}}$, $K_{\text{BOH}}$) in fatal accidental hypothermia are changed compared to the control system (Tables 1 and 2). $c_{\text{A}}$ of erythrocyte membranes decreased compared with control group; however in the case of $c_{\text{B}}$ there were no statistically significant changes. K_AH_ as well as K_BOH_ decreased compared to the control (Table 1). In thrombocytes membranes, it can be observed an increase of $c_{\text{A}}$ value, whereas a decrease of $c_{\text{B}}$ value compared to control. There is also a decrease of *K*_AH_ and *K*_BOH_ values (Table 2).

**Table 1:** The parameters characterizing the equilibria between erythrocytes membrane and the electrolyte solution: total concentrations of acidic (*C*_A_) and basic (*C*_B_) functional groups, association constants with H^+^ and OH^−^ ions (*K*_AH_, *K*_BOH_)

Groups	Parameters			
	*c*_A_ (10^−6^ mol/m^2^)	*c*_B_ (10^−6^ mol/m^2^)	*K*_AH_ (10^2^ m^3^/mol)	*K*_BOH_ (10^7^ m^3^/mol)
Control (Kotyńska et al. 2012)	7.06 ± 0.42	1.54 ± 0.47	3.39 ± 1.12	3.65 ± 0.84
Sudden unexpected death (Kotyńska et al. 2012)	5.34 ± 0.10	1.68 ± 0.08	6.95 ± 0.73	30.7 ± 0.60
Fatal accidental hypothermia	5.62 ± 0.08	1.41 ± 0.11	1.13 ± 0.21	1.01 ± 0.25

**Table 2:** The parameters characterizing the equilibria between thrombocytes membrane and the electrolyte solution: total concentrations of acidic (*C*_A_) and basic (*C*_B_) functional groups, association constants with H^+^ and OH^−^ ions (*K*_AH_, *K*_BOH_)

Groups	Parameters			
	*c*_A_ (10^−6^ mol/m^2^)	*c*_B_ (10^−6^ mol/m^2^)	*K*_AH_ (10^2^ m^3^/mol)	*K*_BOH_ (10^7^ m^3^/mol)
Control (Kotyńska et al. 2012)	3.67 ± 0.79	1.17 ± 0.21	2.81 ± 1.70	2.04 ± 0.59
Sudden unexpected death (Kotyńska et al. 2012)	6.44 ± 0.08	2.71 ± 0.07	4.67 ± 0.43	25.4 ± 0.56
Fatal accidental hypothermia	4.52 ± 0.22	0.85 ± 0.07	2.15 ± 0.25	0.84 ± 0.23

Changes in all determined parameter values ($c_{\text{A}}$, $c_{\text{B}}$, $K_{\text{AH}}$, $K_{\text{BOH}}$) in fatal accidental hypothermia are probably caused by a number of various processes that occur in the human body after death. The consequence of interactions between blood cell membrane components and between them and solution ions is appearance of new groups on membranes surface and disappearance of the existing ones what is responsible for observed changes (Tables 1 and 2).

Fatal accidental hypothermia influences on the blood pH. In the blood samples we collected, pH was around 6.6 (Szeremeta et al. 2015) while the normal blood pH is tightly regulated between 7.35 and 7.45. Deviation from these values is connected with acidosis, which indicates changes in acid—base equilibria occurring in biological membranes and in the end—decrease of membranes surface charge.

Numerous researches have shown interest in postmortem biochemistry related to hypothermia fatalities with several studies pertaining to mainly urine and blood (Hirvonen and Huttunen 1982; Bańka et al. 2013, 2014). Blood ketone bodies have been targeted as being particularly useful in supporting the diagnosis of fatal hypothermia. It was found that deaths due to hypothermia, especially in free-ethanol cases, are characterized by increased ketone levels in blood (Palmiere et al. 2013). Bańka et al. (2014) demonstrated the usefulness of determination of the profile of free fatty acids concentrations as potential markers of hypothermia-related deaths. Beside biochemical characteristics of fatal accidental hypothermia, physicochemical characteristics is important too. The aim of this study was to investigate the usefulness of postmortem physicochemical investigations in the diagnosis of fatal hypothermia. A series of physicochemical parameters were analyzed in order to obtain a more integrated and complete perspective pertaining to the surface charge changes that may occur during hypothermia.

Various scientific disciplines are concerned with research on biomembranes. In this paper, we considered one of the physicochemical aspects of biomembranes related to their structures and electric properties. Understanding these characteristics is invaluable in many areas of biomedical sciences, including forensic medicine.
